# Mechanical and Bond Behavior of a Hybrid Steel–Basalt–Polypropylene Fiber-Reinforced High-Performance Concrete with Steel, GFRP or CFRP Bars

**DOI:** 10.3390/ma19081546

**Published:** 2026-04-13

**Authors:** Piotr Smarzewski

**Affiliations:** Faculty of Civil Engineering and Geodesy, Military University of Technology, 2 Gen. Sylwestra Kaliskiego, 00-908 Warsaw, Poland; piotr.smarzewski@wat.edu.pl

**Keywords:** high-performance concrete (HPC), hybrid fiber reinforcement, steel reinforcement, FRP bars, bond behavior, pull-out test, fracture energy

## Abstract

This study addresses the limited availability of unified experimental datasets comparing ribbed steel and smooth FRP bars embedded in the same hybrid-fiber high-performance concrete (HPC) matrix under identical conditions. It investigates the mechanical and bond behavior of a triple-fiber HPC combining hooked-end steel (ST), basalt (BA), and polypropylene (PP) fibers and reinforced with steel, GFRP, and CFRP bars of identical diameter and embedment. Under a uniform curing regime, the HFRC reached a compressive strength of approximately 82 MPa and exhibited a high fracture energy *G_f_* approximately 3.7 kJ/m^2^ with a stable post-peak response in a notched-beam test, demonstrating effective multi-scale crack bridging within a dense hybrid fiber network. Pull-out tests on 200 mm embedment revealed distinct interfacial mechanisms: ribbed steel developed a pronounced peak bond stress (*τ*_max_ = 13.05 MPa) and the largest bond energy (*G_b_* = 146 N/mm) due to mechanical interlock, whereas smooth GFRP and CFRP showed low *τ*_max_ (=1.46 and 0.78 MPa) and smoothly decaying *τ–s* governed by adhesion–friction with *G_b_* = 3–4 N/mm. A consistent experimental framework enabled direct mechanistic comparison of bond–slip behavior across reinforcement types without confounding matrix or curing variables. Simple constitutive laws calibrated to the experimental *τ–s* curves (ramp–softening for steel and ramp–plateau or exponential for FRP) captured the stiffness, strength, and energy hierarchy with low error. The main contribution of this study lies in providing a configuration-consistent reference dataset and calibrated bond–slip descriptions for hybrid-fiber HPC members reinforced with both steel and FRP bars. The results highlight the role of the hybrid fiber network in improving crack stability and provide design-oriented parameters for anchorage assessment and nonlinear bond–slip modeling. Although the results are based on a limited experimental program, they establish a mechanistically coherent basis for further optimization of hybrid HPC matrices and development of performance-based anchorage formulations in high-performance structural applications.

## 1. Introduction

High-performance concrete (HPC) delivers high compressive strength and durability but remains brittle in tension; hybrid fiber reinforcement is a well-established pathway to enhance crack control and energy absorption across scales (macro steel + meso basalt + micro polypropylene) [1,2,3]. Basalt fibers are particularly attractive due to their high tensile strength, chemical stability, and cost-efficiency, yet their alkali resistance strongly depends on composition and manufacturing processes, which must be considered for long-term stability in cementitious matrices [4]. The present study employs a triple-fiber HPC (steel–basalt–PP) to achieve synergistic improvements in tensile ductility, post-cracking toughness, and interfacial confinement.

In reinforced members, load transfer is governed by the local bond stress–slip (*τ–s*) relationship between the bar and the surrounding concrete. Ribbed steel reinforcement mobilizes mechanical interlock combined with adhesion, whereas smooth glass/carbon fiber-reinforced polymer (GFRP/CFRP) depend primarily on adhesion–friction mechanisms, leading to lower *τ*_max_, larger slip at peak, and a more gradual stress decay [5,6,7]. These mechanisms are highly sensitive to the matrix composition, surface roughness, and embedment length, which together define bond stiffness and failure mode. Recent experimental evidence indicates that fiber reinforcement within HPC or UHPC matrices can substantially alter these mechanisms by improving the integrity of the interfacial transition zone (ITZ) and delaying splitting-type failures [8,9,10,11].

A comprehensive review by Dziomdziora and Smarzewski [12] analyzed over 80 studies on steel, GFRP, CFRP, and hybrid FRP–steel composite bars (SFCBs), summarizing the current understanding of bond, durability, and mechanical performance in fiber-reinforced concretes. The authors emphasized that while FRP bars exhibit excellent corrosion resistance, they suffer from lower stiffness and limited post-cracking ductility compared to steel. Reported elastic moduli range from 40 to 64 GPa for GFRP, from 105 to 175 GPa for hybrid steel–basalt composite bars (SBFCBs), and up to 192 GPa for CFRP, compared to 200 GPa for conventional steel. Hybrid FRP–steel bars effectively combine the ductility of steel with the durability of FRP, showing partially ductile load–slip behavior and delayed failure of the outer FRP shell [12]. However, most available studies evaluate these reinforcement systems in different matrix types, curing regimes, and geometric configurations, which complicates direct mechanistic comparison of bond performance.

Recent studies on the bond behavior of FRP bars embedded in HPC and UHPC matrices [13,14,15] have demonstrated that bond performance is strongly governed by parameters such as bar surface characteristics, embedment length, concrete cover, and matrix composition. In parallel, investigations on hybrid fiber-reinforced cementitious composites [16,17] have confirmed that fiber type, geometry, and dosage significantly influence fracture energy, crack propagation mechanisms, and post-cracking stability.

However, a consistent comparison between these studies remains challenging due to substantial variability in experimental configurations, including differences in specimen geometry, curing regimes, fiber content, and testing methodologies. Although standards such as EN 12390-3 [18], EN 14651 [19], and EN 10080 (Annex D) [20] provide general guidance for mechanical and bond testing, their practical implementation is not fully unified, particularly for pull-out and FRP-related bond tests. As a result, direct comparison and generalization of bond behavior across different studies remain limited.

In this context, pull-out tests on ϕ = 12–19 mm steel bars have reported bond strengths ranging from 20 to 60 MPa, depending on cover, bar geometry, and embedment length. Interestingly, increasing steel fiber content from 1% to 2% may reduce the measured bond by 24%, as excessive fiber dosage disrupts matrix homogeneity and local confinement [8]. Other studies have shown that silica-fume-based UHPC repair mortars containing steel and basalt fibers produce denser ITZs and higher adhesion strength due to refined microstructure, as verified by SEM, XRD, and MIP analyses [21,22,23]. These observations confirm that fiber synergy (particularly steel and basalt combinations) can influence both matrix toughness and bar bond behavior.

While hybrid fiber reinforcement in HPC has been extensively studied from the perspective of compressive, tensile, and flexural performance, its interaction with fundamentally different reinforcement mechanisms (mechanical interlock vs. adhesion–friction) under unified experimental conditions has not been systematically addressed.

Despite extensive research on steel–HPC/UHPC and FRP–concrete bond behavior, a systematic comparison of ribbed steel and smooth FRP bars embedded in the same hybrid-fiber HPC matrix, tested under identical diameter, embedment, and curing conditions, remains scarce. In particular, complete *τ–s* curves combined with fracture-energy-based interpretation (*G_f_*, *G_b_*) for such unified systems are rarely reported.

To address these gaps, this study investigates a triple-fiber HPC matrix (steel–basalt–PP) combined with steel, GFRP, or CFRP reinforcement, generating a consistent dataset comprising compressive, splitting and flexural strengths, fracture energy, and pull-out responses. The analysis integrates complete τ–s and load–deflection curves with energy-based interpretations (*G_f_*, *G_b_*), supported by macro-level observations of failure modes. Particular attention is given to the distinct interfacial mechanisms (mechanical interlock versus adhesion–friction) arising from the stiffness hierarchy of the reinforcing bars (*E*_steel_ = 200 GPa > *E*_CFRP_ = 160 GPa > *E*_GFRP_ = 50 GPa) and interpreted in relation to current anchorage and development-length provisions of EN 1992-1-1, EN 1992-4, ACI 318-19, ACI 440.1R-15, and the fib Model Code 2010 [24,25,26,27,28].

Accordingly, the novelty of the present study lies in delivering a configuration-consistent experimental comparison of ribbed steel and smooth FRP bars embedded in the same hybrid-fiber HPC matrix under identical geometric and curing conditions, integrating complete *τ–s* characterization with fracture-energy-based interpretation and calibrated bond–slip descriptions suitable for structural modeling and anchorage assessment.

The present study is intentionally designed as a preliminary and exploratory investigation, aimed at providing a mechanistically consistent reference dataset for comparing bond behavior and fracture-related responses of different reinforcement types embedded in the same hybrid-fiber HPC matrix under identical conditions.

## 2. Experimental Program

### 2.1. Materials

#### 2.1.1. Cement and Aggregates

The binder used in the experimental program was Portland cement CEM I 42.5 R, manufactured by Cemex Polska (Cement Plant, Chełm, Poland). The cement conforms to EN 197-1 specifications [29] and is classified as a high-early-strength Portland cement with rapid hydration and moderate fineness (Table 1).

The coarse aggregate was crushed granite supplied by Granit Strzegom S.A., originating from the Strzegom–Sobótka granitoid massif located in Lower Silesia (Poland). Two fractions were employed: 2–8 mm and 8–16 mm. According to the certificate of the manufacturer, the aggregate exhibits a bulk density of 2625–2630 kg/m^3^, an open porosity below 1%, a water absorption of =0.3%, and a compressive strength of 185–190 MPa. The fine aggregate was natural quartz sand (FA) with a maximum particle size of 2 mm and a fineness modulus of 1.84, ensuring dense packing and a smooth surface texture favorable for fiber distribution and bond development.

#### 2.1.2. Admixture

A high-range water-reducing admixture of type Glenium SKY 591 (FM)/(BV), produced by BASF Construction Chemicals, Ludwigshafen, Germany was used to achieve high flowability at a low water-to-cement ratio. This polycarboxylate-ether (PCE)-based product provides efficient cement particle dispersion and stabilization of the suspension, reducing internal friction and enhancing homogeneity. The superplasticizer is a brown liquid with a density of 1.07 ± 0.02 g/cm^3^ and a pH value of 6.5 ± 1.0 at 20 °C. Chloride and alkali contents do not exceed 0.1% and 1.5% by mass, respectively. The recommended dosage is 0.2–3.0% of cement mass. In this study, a dosage of 2.7% was used. The admixture enabled maintaining a low water-to-cement ratio (w/c = 0.28), suitable for the target HPC strength level.

#### 2.1.3. Fibers

**Figure 1:**
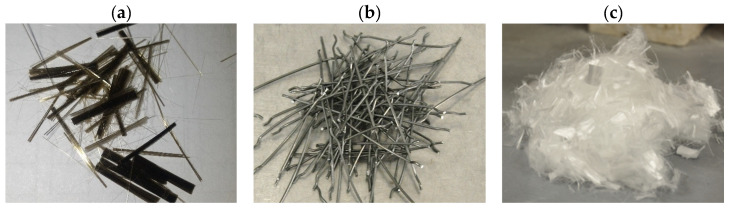
Fibers used in the study: (**a**) basalt fibers (BF); (**b**) steel fibers (ST); (**c**) polypropylene fibers (PP).

Three types of fibers were used to produce a hybrid fiber-reinforced high-performance concrete: hooked-end steel fibers (ST), basalt fibers (BF), and fibrillated polypropylene fibers (PP) (Table 2, Figure 1).

Steel fibers (ST) of type BAUMIX^®^ (Bautech^®^, Piaseczno, Poland) had a hooked-end shape, a 50 mm length, and a 1.0 mm diameter, with a density of 7.8 g/cm^3^, an elastic modulus of 200 GPa, and a tensile strength of 1100 MPa. Basalt fibers (BF) were 12 mm long chopped strands consisting of 13 µm-diameter filaments, flattened into bundles approximately 1 mm wide. Their physical properties included a density of 2.7 g/cm^3^, an elastic modulus of 70 GPa, a tensile strength of 1700 MPa, and an elongation at break of 2.5%. Polypropylene fibers (PP) of type BAUCON^®^ (Bautech^®^, Poland) were fibrillated, 12 mm long, and 0.03 mm in nominal diameter, with a density of 0.9 g/cm^3^, an elastic modulus of 3.5 GPa, and a tensile strength of 350 MPa.

The combined use of steel, basalt, and polypropylene fibers was designed to exploit multi-scale reinforcement synergy within the high-performance concrete matrix. Steel fibers provide macro-crack bridging capacity and contribute to post-cracking toughness and energy dissipation. Basalt fibers, due to their intermediate stiffness and smaller diameter, enhance tensile resistance and control micro- to meso-scale crack propagation, while polypropylene fibers mitigate plastic shrinkage cracking and improve early-stage crack dispersion.

The selection of fiber lengths was consistent with this multi-scale concept. The 50 mm hooked-end steel fibers were adopted to bridge macro-cracks and sustain load transfer at larger crack openings, whereas the shorter 12 mm basalt and polypropylene fibers act at smaller crack-width regimes, limiting crack initiation and propagation at earlier stages. This differentiated geometry enables complementary crack-control mechanisms and promotes more uniform stress redistribution within the hybrid-fiber HPC matrix.

#### 2.1.4. Reinforcing Bars

**Figure 2:**
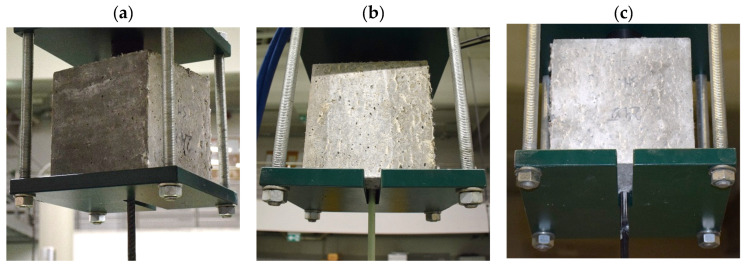
Pull-out test setup for 200 × 200 × 200 mm hybrid-fiber HPC cubes: (**a**) specimen with steel reinforcing bar; (**b**) specimen with a GFRP bar; (**c**) specimen with a CFRP bar. All bars had a nominal diameter of 12 mm and identical embedment lengths.

Three types of reinforcing bars were investigated: ribbed steel, glass fiber-reinforced polymer (GFRP), and carbon fiber-reinforced polymer (CFRP) bars, all with a nominal diameter of 12 mm (Figure 2).

The steel bars were B500SP with ribbed geometry ensuring mechanical interlock with the surrounding matrix. The mean yield and ultimate tensile strengths obtained from direct tension tests on bars cut from the same batch were 605 MPa and 691 MPa, with an elastic modulus of 203 GPa and an ultimate strain of approximately 33%. The measured rib face angles ranged between 37° and 73°, and the distance between ribs varied from 3 to 6 mm. The GFRP and CFRP bars had nominal diameters of 12 mm and smooth cylindrical surfaces, relying primarily on adhesion and friction against the hybrid fiber-reinforced concrete (HFRC) matrix. It is acknowledged that FRP bars used in structural practice are often sand-coated, ribbed, or otherwise surface-treated to enhance mechanical interlock [30]. The inclusion of FRP bars was motivated by their corrosion resistance and potential use in hybrid reinforcement strategies in aggressive environments. The use of smooth bars in this study was intentional, aiming to isolate the fundamental adhesion–friction bond mechanism and to provide a conservative (lower-bound) reference case for comparison with ribbed steel reinforcement under identical matrix and curing conditions.

Their main mechanical properties (as provided by the manufacturer) are summarized in Table 3.

The significantly different stiffness, surface geometry, and failure modes of these bars are expected to have a direct influence on the interfacial bond behavior described in Section 3.3.

### 2.2. Experimental Program

#### 2.2.1. Mix Composition

The hybrid fiber-reinforced high-performance concrete was designed using a low water-to-cement ratio (w/c = 0.28) and a combination of three types of fibers: steel, basalt, and polypropylene, in order to obtain a dense high-performance matrix with reduced porosity, enhanced mechanical strength, and improved fiber–matrix and bar–matrix interaction. The mixture composition per cubic meter is presented in Table 4. Portland cement CEM I 42.5 R (Cemex Polska, Chełm Plant) was used as the primary binder. Crushed granite aggregate (2–8 mm and 8–16 mm fractions) originated from Strzegom quarry, while fine quartz sand (0–2 mm) served as the fine aggregate. Tap water was used for mixing, and a polycarboxylate-based superplasticizer (Glenium SKY 591, BASF, Ludwigshafen, Germany) was applied to ensure adequate workability and uniform fiber dispersion.

The steel (ST), basalt (BF), and polypropylene (PP) fibers were introduced at total dosages of 25.7, 8.7, and 3.0 kg/m^3^, respectively, corresponding to a combined volumetric fraction of approximately 1% (≈0.33% of each fiber type). The selected fiber dosage was based on a balance between mechanical enhancement and workability constraints typical for high-performance concrete. A total volumetric fraction of approximately 1% was adopted to ensure effective crack bridging while maintaining adequate flowability and fiber dispersion. The approximately equal volumetric contribution of each fiber type was chosen to promote multi-scale reinforcement synergy without allowing one fiber class to dominate the composite response. Higher total fiber contents were intentionally avoided to prevent excessive stiffness reduction, fiber clustering, and deterioration of fresh mix rheology, which could adversely affect bond behavior and matrix homogeneity. The mix was produced in a high-shear pan mixer to achieve homogeneous dispersion of fibers and to minimize air entrainment.

The relatively high cement content was intentionally adopted to ensure a dense and mechanically stable reference matrix with minimal variability in bond-related parameters. The primary objective of the present study was a mechanistic comparison under controlled conditions rather than environmental optimization. Supplementary cementitious materials (SCMs) were not incorporated in order to avoid additional variables affecting matrix chemistry and interfacial transition zone (ITZ) behavior. The integration of SCM-based binders in hybrid-fiber HPC systems remains an important direction for future research.

#### 2.2.2. Specimen Preparation and Pull-Out/Flexural Test Setup

A series of specimens were prepared for the mechanical and bond tests, following relevant European and Polish standards.

After demolding at 24 ± 2 h, all specimens were cured in water tanks at 20 ± 2 °C until the day of testing, in accordance with PN-EN 12390-2:2019-07 [31]. All mechanical and bond tests were conducted after 28 days of water curing.

Compressive strength tests were performed on six cubic specimens (150 × 150 × 150 mm) in accordance with PN-EN 12390-3:2019-07 [18], which specifies standardized procedures for determining compressive strength of hardened concrete under controlled loading conditions. The tests were carried out using an Advantest 9 universal testing machine (CONTROLS, Milan, Italy) with a maximum load capacity of 3 MN with a load rate of 0.6 ± 0.2 MPa/s, as specified by the standard.

Splitting tensile strength was determined on the same type and number of cubic specimens in accordance with PN-EN 12390-6:2011 [32]. The applied load was distributed along two opposite lines using steel bearing strips to ensure uniform stress distribution. The tests were carried out on the same type of universal testing machine.

Flexural strength tests were performed on twelve prismatic beams (100 × 100 × 500 mm) according to PN-EN 12390-5:2019-08 [33]. Six beams were tested under three-point bending, and six under four-point bending configurations, with a span length of 300 mm and a constant deflection control rate of 0.05 mm/min, to capture both the strength and ductility effects of hybrid fiber reinforcement. The four-point bending setup had a load spacing of 100 mm between the two applied forces. The tests were carried out using a Walter + Bai universal testing machine (Walter + Bai AG, Löhningen, Switzerland).

Fracture behavior of the hybrid-fiber HPC mixture was analyzed using a single notched beam specimen (80 × 150 × 700 mm, notch depth = 50 mm) subjected to three-point bending using an MTS 319.25 servo-hydraulic testing machine (MTS Systems Corporation, Eden Prairie, MN, USA) with a maximum load capacity of 250 kN, following the procedure conceptually based on RILEM TC 50-FMC [34] and EN 14651:2005+A1:2007 [19] which provides a standardized methodology for evaluating flexural performance and post-cracking behavior of fiber-reinforced concrete. The support span was 550 mm, and the displacement-controlled loading rate was identical to that used for the unnotched beams. Load–deflection data were recorded for fracture energy evaluation. The objective of this test was exploratory and mechanistic, focusing on the characterization of fracture response under controlled conditions. Therefore, statistical variability cannot be assessed based on a single specimen.

Bond tests were conducted on three cubic specimens with dimensions of 200 × 200 × 200 mm (one per bar type: steel, GFRP, and CFRP), each containing one centrally embedded reinforcing bar. The primary objective was a controlled comparative assessment of interfacial mechanisms under identical matrix composition, curing regime, embedment length, and loading configuration. The results should therefore be interpreted as configuration-specific and preliminary rather than statistically generalizable. The limited number of specimens reflects the exploratory nature of the study and is intended to enable a controlled mechanistic comparison between reinforcement types rather than a statistical evaluation of variability.

**Figure 3:**
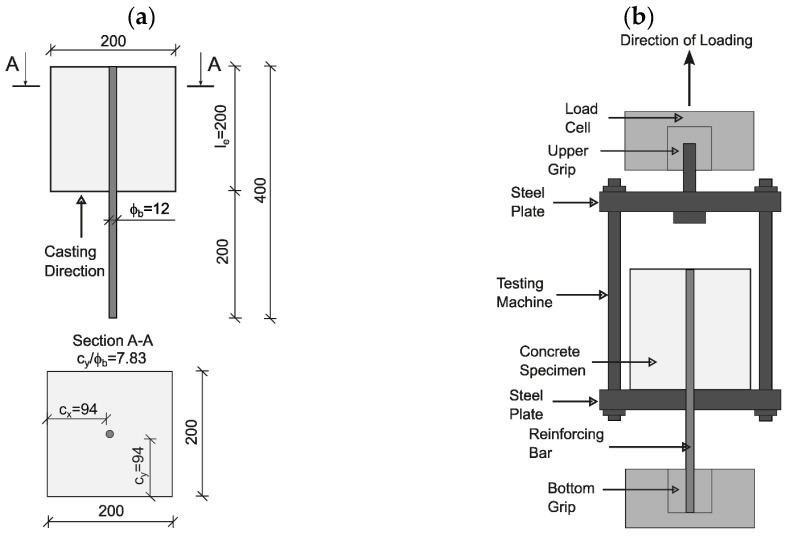
Geometry and testing setup for pull-out tests: (**a**) specimen dimensions and reinforcement placement; (**b**) testing assembly with vertical loading configuration.

The test setup followed the general concept of the EN 10080 (Annex D) recommendation [20], which provides guidelines for assessing bond behavior between reinforcement and concrete using pull-out test configurations using a monotonic loading rate under displacement control. The embedment length and loading configuration are detailed in Figure 3.

All the specimens were cast with the reinforcing bar positioned vertically in the center of the mold (Figure 3a). The pull-out tests were performed using the same MTS machine as in the fracture test (Figure 3b). The load was applied monotonically under displacement control at a rate of 1 mm/min until bond failure or complete bar slippage.

Due to the absence of dedicated slip-measuring instrumentation, the relative displacement between the bar and concrete was approximated from the actuator displacement of the testing machine. A correction for system compliance was applied during post-processing to account for machine and fixture deformation. It should be noted that this approach provides an effective system-level slip measure rather than a direct local interface displacement. Consequently, the initial bond stiffness values (*k*_0_) reported herein should be interpreted as effective comparative parameters rather than absolute interface stiffness. While this method enables consistent comparison between steel, GFRP, and CFRP bars under identical conditions, future investigations incorporating direct slip measurements (e.g., LVDTs or digital image correlation) would reduce uncertainty in the early-slip regime and improve stiffness quantification. In particular, the use of actuator displacement may lead to an overestimation of slip in the initial loading stage due to machine compliance and seating effects, which can result in an underestimation of the true initial bond stiffness.

It should be noted that the direct pull-out test provides a simplified representation of bond behavior under predominantly axial loading and does not fully reproduce the stress state occurring in flexural members, where bond is influenced by cracking patterns, transverse stresses, and cover confinement. Therefore, the obtained *τ–s* relationships should be interpreted as local interfacial responses rather than direct predictors of structural anchorage performance.

The bond stress was computed as

(1) $$\tau =\frac{P}{\pi dl_{e}}$$

where *P* is the applied load, *d* is the bar diameter, and *l_e_* is the embedment length (200 mm).

### 2.3. Data Processing

All experimental data were processed to obtain the representative mechanical and bond parameters of the hybrid fiber-reinforced HPC mixture. For each test series, the results were statistically evaluated using the mean value, standard deviation (SD), coefficient of variation (COV), and 95% confidence interval (CI) calculated via a bootstrap resampling method (10,000 iterations). Outlier values, if any, were verified using the Iglewicz–Hoaglin robust criterion based on modified z-scores.

The normalization of strength parameters was adopted to facilitate a comparison between mechanical and bond test results. The splitting tensile strength (*f*_*ct*,*sp*_) and flexural strength (*f*_*ct*,*flex*_) were expressed relative to the compressive strength (*f_c_*), whereas the maximum bond stress (*τ_max_*) was normalized with respect to √*f_c_*, following the convention in anchorage and development length formulations.

For the notched beam test, the fracture energy (*G_f_*) was calculated using the work-of-fracture method, integrating the load–deflection curve:

(2) $$G_{f}=\frac{\int P\delta d\delta }{b\left(h-a_{0}\right)}$$

where *P* is the load, *δ* is the midspan deflection, *b* and *h* are the beam width and height, respectively, and *a*_0_ is the initial notch depth. This approach provides an effective measure of energy dissipation and crack propagation resistance of the hybrid fiber-reinforced matrix.

From the pull-out tests, the bond stress–slip (*τ–s*) curves were derived using Equation (1). The corresponding slip *s* was evaluated as the displacement at the loaded end of the bar, as measured by the testing system.

The main bond parameters were obtained as follows: *τ*_max_—maximum bond stress, *s*(*τ*_max_)—slip corresponding to *τ*_max_, *k*_0_—initial bond stiffness, determined as the tangent slope of the *τ–s* curve at the origin, *G_b_*—total bond energy, representing the area under the *τ–s* curve up to complete bar pull-out or debonding:

(3) $$G_{b}=\int_{0}^{s_{\max}}\tau \left(s\right)ds$$

All datasets were post-processed using consistent statistical and numerical procedures to ensure the reproducibility and reliability of results. The *τ–s* and load–deflection curves are presented in Section 3 together with comparative analyses of bond and fracture energies.

## 3. Results and Discussion

### 3.1. Mechanical Properties

The compressive, splitting tensile, and flexural strengths of the hybrid-fiber HPC mixture were determined after 28 days of water curing. Table 5 summarizes the mean values, standard deviation (SD), coefficient of variation (COV), and 95% confidence intervals (CI). The mixture exhibited a high compressive strength (approximately 82 MPa) and balanced tensile response, reflecting the effective hybridization of steel, basalt, and polypropylene fibers. The differences between three- and four-point bending tests were consistent with typical stress redistribution patterns observed in fiber-reinforced concretes, where the higher bending span in the 4-pt configuration results in slightly lower nominal tensile stress.

**Figure 4:**
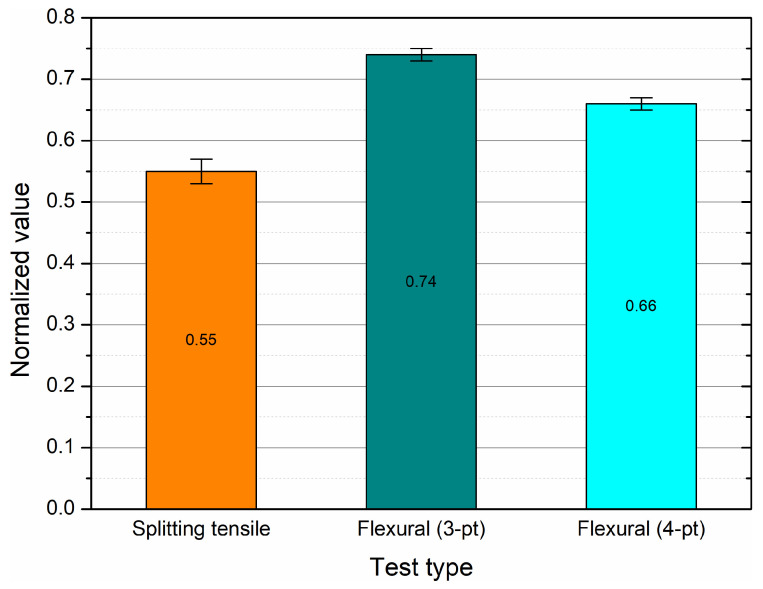
Normalized splitting tensile and flexural strengths of the hybrid-fiber HPC mixture (*f*_*ct*,*sp*_/√*f_c_* and *f*_*ct*,*flex*_/√*f_c_*) with 95% confidence intervals.

A normalized comparison of tensile to compressive strength ratios (*f*_*ct*,*sp*_/√*f_c_* and *f*_*ct*,*flex*_/√*f_c_*) is shown in Figure 4. The observed relationships fall within the upper range of HPC and hybrid-fiber HPC reported in literature, indicating strong fiber–matrix interaction and reduced brittleness under flexural loading.

**Figure 5:**
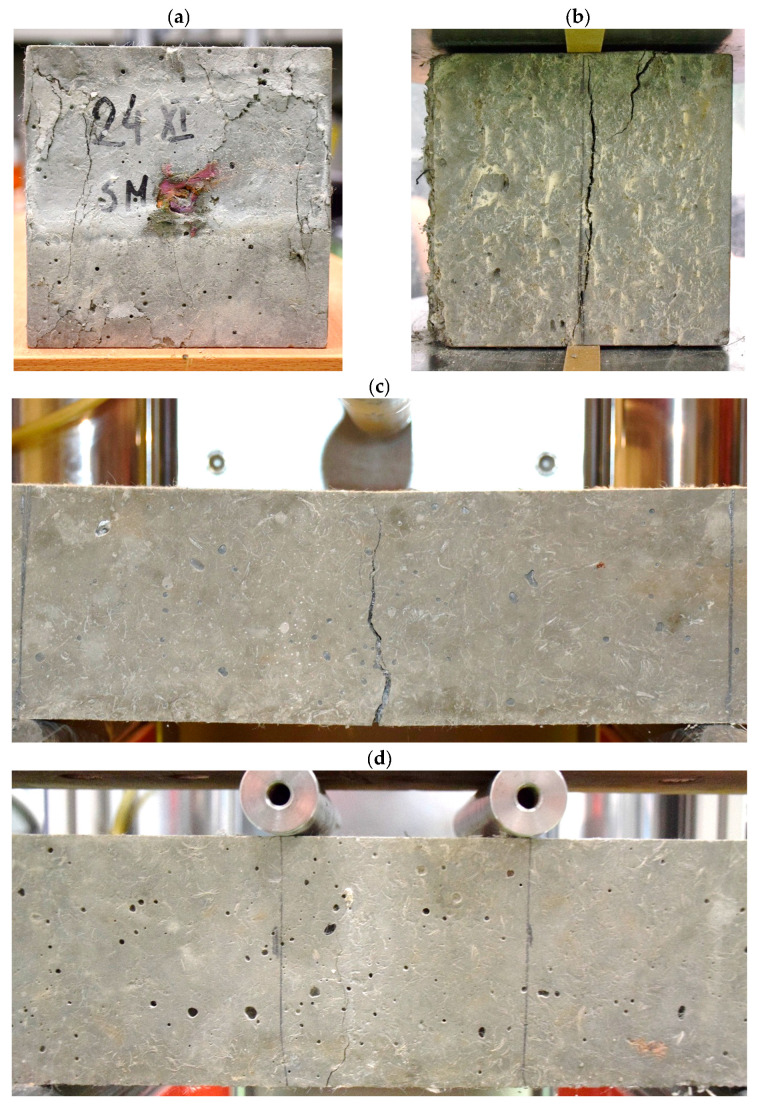
Failure modes observed during mechanical testing of hybrid-fiber HPC specimens: (**a**) compressive test cube; (**b**) splitting tensile specimen; (**c**) three-point bending beam; (**d**) four-point bending beam.

The typical failure patterns observed during testing are shown in Figure 5. The compressive specimens exhibited uniform crushing without explosive failure (Figure 5a), indicating stable load transfer through the dense hybrid-fiber HPC matrix. In splitting tensile tests, a single dominant crack propagated vertically along the loading plane (Figure 5b). Flexural specimens tested under three- and four-point bending developed localized cracks in the mid-span region, as shown in Figure 5c,d, demonstrating cohesive crack propagation and efficient fiber bridging across the fracture zone.

### 3.2. Fracture Behavior

**Figure 6:**
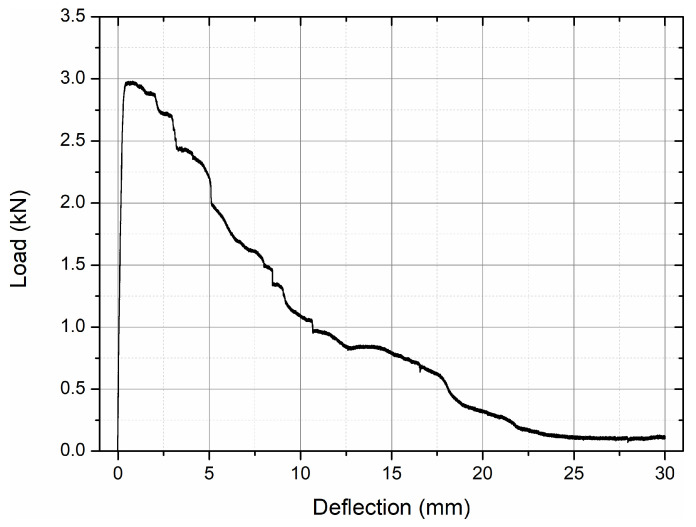
Notched beam (HPC with ST/BA/PP fibers) under three-point bending: load–deflection curve P–δ.

The fracture response of the hybrid-fiber HPC mixture was assessed on a single notched beam (80 × 150 × 700 mm) under three-point bending (see Figure 6). The support span was *L* = 550 mm, and the notch depth was *a*_0_ = 50 mm. Load–deflection data (*P–δ*) were recorded at mid-span under displacement control using the same testing press as in the pull-out experiments. Crack mouth opening displacement (*CMOD*) was not measured. Therefore, fracture parameters are reported on the basis of the work-of-fracture approach using *P–δ*. This choice is consistent with methods recommended for notched beams with the caveat that direct comparison to *CMOD*-based post-cracking indices should be made cautiously.

It should be emphasized that the fracture response reported herein corresponds to a single representative specimen tested under strictly controlled conditions. The purpose of this experiment was to obtain a mechanistically consistent characterization of the fracture process in the hybrid-fiber HPC matrix rather than to derive statistically distributed fracture parameters. Accordingly, the reported values should be interpreted as configuration-specific and indicative of the material behavior under the adopted testing setup.

The main fracture parameters derived from the load–deflection curve are summarized in Table 6. The fracture energy *G_f_* was computed via the work-of-fracture method from Equation (2), where *b* = 80 mm, *h* = 150 mm, and *a*_0_ = 50 mm. The integral ∫*Pdδ* was evaluated as the trapezoidal area under the measured *P–δ* curve up to the ultimate deflection *δ_max_*. The peak load *P_max_* and corresponding deflection *δ*(*P_max_*) were extracted to characterize the onset of unstable cracking. Since CMOD was not instrumented, the commonly reported residual strengths defined at CMOD = 0.5–1.5 mm in EN 14651 [19] are not available. Instead, indicative residual load ratios were derived at corresponding mid-span deflection levels *P*(*δ* = 0.5, 1.0, 1.5 mm)/*P_max_*. These residual load ratios represent the normalized load-carrying capacity of the specimen at selected post-peak deflection levels relative to the peak load, i.e., the ratio between the load at a given deflection and the maximum load (*P*/*P_max_*).

It must be emphasized that these values are non-standard, deflection-based surrogate parameters and are not directly comparable to CMOD-based residual strengths. Mid-span deflection incorporates global beam deformation in addition to crack opening displacement, which may lead to an apparent stabilization of early post-peak response and potentially higher residual ratios compared to CMOD-controlled measurements. Accordingly, the reported residual load ratios are intended solely for internal characterization of post-peak ductility and fiber-bridging behavior within the present experimental configuration.

Residual load ratios remained close to unity up to *δ* = 1.5 mm, indicating stable post-peak bridging. A significant reduction (below 0.9*P_max_*) occurred beyond *δ* ≈ 2.9 mm, marking the onset of macrocrack propagation. The obtained fracture energy (*G_f_* ≈ 3.7 kJ/m^2^) lies well above the typical range reported for conventional high-performance concrete without fibers (commonly 100–300 J/m^2^) and exceeds values frequently reported for single-fiber steel-reinforced systems at comparable strength levels (approximately 1.5–3.0 kJ/m^2^, depending on fiber dosage and geometry). This indicates a significant enhancement of post-cracking energy dissipation capacity associated with the synergistic action of macro steel fibers, meso-scale basalt fibers, and micro polypropylene fibers.

From a multi-scale perspective, steel fibers primarily contribute to macro-crack bridging and resistance to crack opening at larger deflections, basalt fibers enhance tensile resistance and microcrack stabilization within the fracture process zone, while polypropylene fibers improve crack dispersion at early stages and reduce localization. The combined action results in delayed crack coalescence and extended stable softening, as reflected in the near-unity residual load ratios up to δ = 1.5 mm and the high fracture energy (*G_f_* ≈ 3.7 kJ/m^2^).

**Figure 7:**
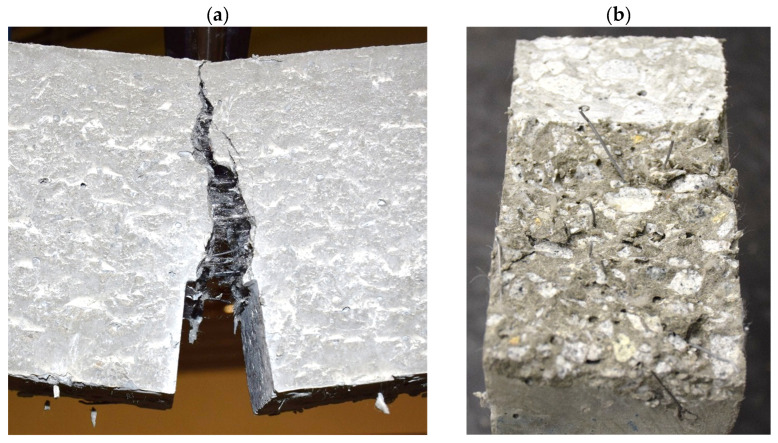
Visual documentation of fracture: (**a**) localized crack at the notch immediately before termination of loading (macro-bridging visible); (**b**) fracture surface with evident hooked-steel fiber pull-out and dispersed basalt/PP fibers.

Typical crack localization at the notch root was observed with progressive fiber bridging (Figure 7a), followed by a stable pull-out of hooked steel fibers and partial rupture/pull-out of basalt and polypropylene fibers across the fracture plane (Figure 7b). The crack path remained essentially vertical through the ligament, indicating a dominant Mode-I separation. The residual load in the softening branch was governed by fiber bridging and extraction, consistent with the hybrid steel–basalt–PP system.

Although CMOD was not directly measured, the deflection-based residual ratios provide a consistent internal comparison of post-peak response for the tested configuration. Future investigations including direct CMOD instrumentation would enable alignment with standardized residual strength classifications.

### 3.3. Bond Behavior

**Figure 8:**
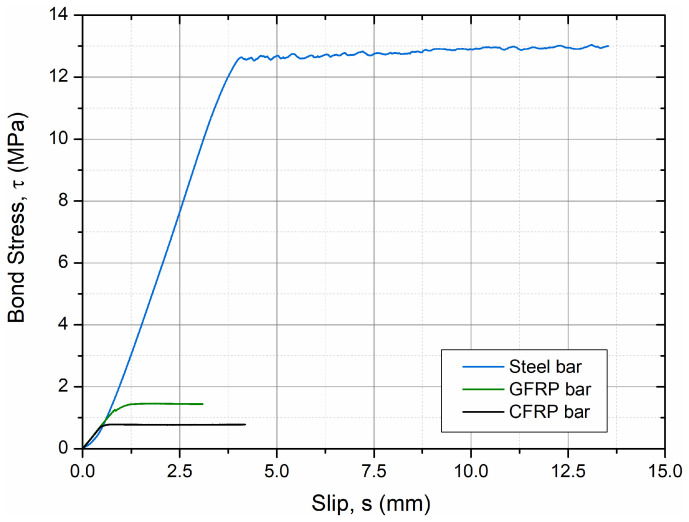
Experimental *τ–s* curves for steel, GFRP, and CFRP bars.

Figure 8 presents the experimentally obtained bond stress–slip (*τ–s*) curves for the three bar types (steel, GFRP, and CFRP) embedded in hybrid-fiber HPC. The curves demonstrate distinctly different interfacial behaviors reflecting the surface morphology and mechanical interlock of each reinforcement type. The smooth-surfaced GFRP and CFRP bars exhibited a monotonic increase in bond stress followed by gradual softening without a clear peak, which is characteristic of an adhesion–friction bond governed by interface degradation. In contrast, the ribbed steel bar showed a well-defined maximum bond stress (*τ_max_* = 13.05 MPa) followed by a descending branch associated with progressive rib crushing and pull-out (Figure 8). The substantially higher bond stress of the ribbed steel bar is primarily attributed to mechanical interlock between ribs and the surrounding matrix, whereas the smooth FRP bars rely predominantly on adhesion–friction mechanisms. Although this results in significantly lower peak bond stresses for GFRP and CFRP, FRP reinforcement remains relevant in durability-driven applications and hybrid reinforcement strategies where corrosion resistance and long-term performance are critical considerations.

The main bond parameters obtained from the experimental *τ–s* relationships are summarized in Table 7. The highest *τ_max_* and bond energy (*G_b_* = 145.94 N/mm) were recorded for the steel bar, confirming the strong mechanical interlock between ribs and the hybrid-fiber HPC matrix. The GFRP and CFRP bars, due to their smooth surface finish and limited chemical adhesion, reached only 1.46 MPa and 0.78 MPa, respectively, with corresponding *G_b_* values below 4 N/mm. The calculated initial bond stiffness (*k*_0_) also varied significantly, highest for the CFRP bar (11.65 MPa/mm) due to its elastic response and lowest for the ribbed steel bar (0.44 MPa/mm), where slip localizes through rib deformation.

The normalized bond strength (*τ_max_*/√*f_c_*) values for the FRP bars (≈0.09–0.16) fall within the range reported for GFRP and CFRP–concrete interfaces in high-performance matrices [35,36,37], while the steel bar exhibited a much higher normalized value (>0.6), consistent with previous findings for ribbed steel reinforcement in HPC [38,39]. The substantially higher bond energy of the ribbed steel bar suggests the activation of pronounced mechanical interlock combined with confinement effects within the hybrid-fiber HPC matrix. This interpretation is consistent with the macroscopic *τ–s* response and observed surface damage patterns, as well as with previous microstructural investigations of fiber-reinforced HPC systems reported in the literature [8,9,10,11,21,22,23]. However, it should be noted that the proposed interpretation of ITZ refinement and fiber–matrix interaction is based on macroscopic experimental observations and literature evidence, as no direct microstructural characterization (SEM or XRD) was performed in the present study.

**Figure 9:**
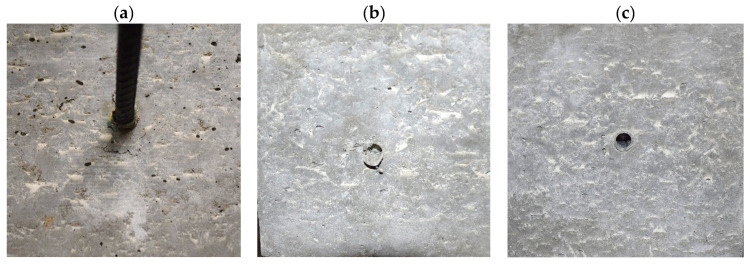
Post-test appearance of pull-out specimens: (**a**) steel; (**b**) GFRP; (**c**) CFRP bars.

Macro-photographs of the tested specimens are shown in Figure 9a–c. The pull-out response was governed primarily by bar extraction in all cases; however, the extent of observable surface damage differed between reinforcement types. For the ribbed steel bar, the specimen exhibited only localized surface distress near the loaded end (small chipping/abrasion around the exit zone), which is consistent with mechanical interlock and local rib-induced bearing stresses. The relatively long embedment length (200 mm in all specimens) likely distributed bond stresses along the anchorage zone and limited the development of pronounced splitting or cone-type surface cracking. In contrast, both FRP bars were completely pulled out without visible macrocracking of the surrounding concrete, indicating an adhesion–friction controlled interfacial debonding mechanism. The absence of splitting cracks in the FRP specimens is consistent with the smooth bar surface and the low bond stress level mobilized in the present configuration.

## 4. Modeling of Bond Behavior

### 4.1. Constitutive Models

The bond behavior between the reinforcing bar and the hybrid fiber-reinforced HPC matrix was modeled using distinct constitutive laws for ribbed steel bars and smooth FRP bars. These reinforcement types exhibit different interfacial mechanisms: mechanical interlock in the case of steel bars and adhesion–friction for FRP bars. The adopted formulations are phenomenological calibrations of the experimentally measured *τ–s* curves and are not intended to replace established code-based bond laws. Instead, they provide simplified but mechanically interpretable descriptions suitable for comparative analysis and nonlinear numerical implementation.

For steel bars, the experimental *τ–s* response demonstrated an initial linear ascending branch, followed by a transition into a region of reduced stiffness. The bond stress continued to increase after yielding, without a distinct plateau or degradation. This behavior, consistent with mechanical interlock and confinement effects, was modeled using a ramp–softening law:

(4) $$\tau \left(s\right)=\left\{\begin{array}{ll} k_{0}s, & 0\leq s\leq s_{1}\\ \tau_{1}+k_{1}\left(s-s_{1}\right), & s>s_{1} \end{array}\right.\;$$

where *τ*(*s*) is the bond stress [MPa], *s* is the slip [mm], *k*_0_ is the initial bond stiffness [MPa/mm], *τ*_1_ = *k*_0_*·s*_1_, and *k*_1_ is the secondary stiffness [MPa/mm].

The ramp–softening formulation conceptually aligns with classical interlock-based bond models, in which bond degradation follows progressive rib bearing and micro-cracking in the surrounding matrix. Similar ascending–descending trends have been reported in nonlinear bond–slip formulations for ribbed reinforcement in high-performance concretes. Although the proposed bond–slip formulations were calibrated for the present experimental configuration, they reflect general trends observed in fiber-reinforced systems, where interfacial behavior can be idealized as a combination of stiffness-controlled ascending response and mechanism-dependent post-peak evolution. This provides a simplified but physically consistent framework for comparative analysis and potential implementation in nonlinear structural modeling.

For FRP bars (GFRP and CFRP), the *τ–s* curves also showed an initial linear increase but were followed by a bond stress plateau, indicating sustained frictional resistance rather than interfacial breakdown. These behaviors were modeled using a ramp–plateau law:

(5) $$\tau \left(s\right)=\left\{\begin{array}{ll} k_{0}s, & 0\leq s\leq s_{1}\\ \tau_{\max}, & s>s_{1} \end{array}\right.\;$$

where *s*_1_ is the slip at which *τ* reaches its maximum value and remains constant, and *τ*_max_ = *k*_0_*·s*_1_.

The ramp–plateau representation reflects the adhesion–friction dominated behavior commonly reported for smooth FRP bars, where the absence of mechanical interlock limits post-peak softening and prevents pronounced stress degradation. In the present hybrid-fiber HPC matrix, this resulted in relatively stable stress transfer up to complete pull-out, without splitting or abrupt bond failure. However, due to the low bond stress level and limited interfacial confinement, the overall energy dissipation remained small, and the bars were extracted at comparatively low slip values, indicating full interfacial debonding governed primarily by adhesion and friction.

### 4.2. Parameter Fitting

Model parameters were fitted to experimental *τ–s* data using a nonlinear least-squares approach. The fitting quality was evaluated using the coefficient of determination (R^2^) and root mean square error (RMSE). Table 8 summarizes the best-fit parameters. The ramp–softening model was applied to the ribbed steel bar, and the ramp–plateau model was used for both FRP bars (GFRP and CFRP).

**Figure 10:**
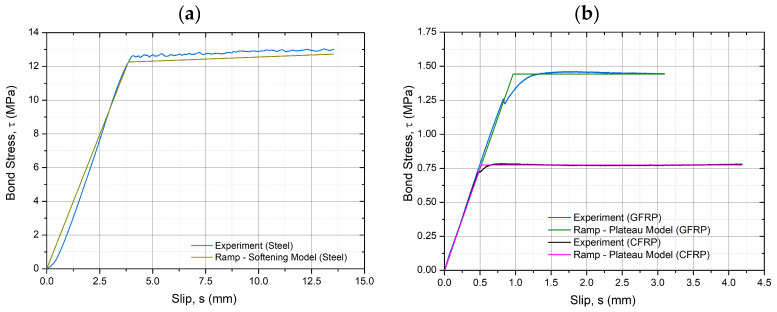
Comparison of experimental and modeled *τ–s* relationships: (**a**) steel bar (ramp–softening model); (**b**) FRP bars (ramp–plateau model).

The comparison between experimental and modeled *τ–s* relationships is presented in Figure 10, showing excellent agreement for both model types.

Accordingly, the proposed τ–s descriptions should be interpreted as configuration-specific and comparative rather than universally generalizable, serving primarily to highlight relative differences in interfacial mechanisms among the investigated reinforcement systems.

### 4.3. Derived Parameters and Implications

The bond energy *G_b_* was computed by numerically integrating the *τ–s* curve from Equation (3). The results confirmed the substantially higher bond energy of the steel bar (~146 N/mm) compared to GFRP (~3.8 N/mm) and CFRP (~3.0 N/mm). This indicates that the ribbed steel bar mobilized strong mechanical interlock and confinement through the hybrid fiber network, while FRP bars relied exclusively on adhesion and friction, with much earlier debonding and limited energy absorption. The marked contrast in bond energy highlights the fundamentally different force-transfer mechanisms: energy dissipation in steel is governed by progressive interlock mobilization along the embedment length, whereas in FRP bars it is limited to surface adhesion and frictional resistance. This distinction has direct implications for anchorage design and crack control in hybrid-fiber HPC members.

Normalized bond strengths *τ_max_*/√*f_c_* ranged from 0.09 (CFRP) to 1.44 (steel), aligning with values reported in [35,37]. To further interpret anchorage performance, the effective anchorage length *l*_*b*,*eff*_ was calculated using the following design-level expressions:

(6) $$l_{b,eff}=\frac{\phi f_{y}}{4\tau_{\max}}\;(\mathrm{for}\;\mathrm{steel})\;\mathrm{or}\;l_{b,eff}=\frac{\phi f_{u}}{4\tau_{\max}}\;(\mathrm{for}\;\mathrm{FRP})$$

where *ϕ* = 12 mm is the bar diameter (common to all specimens), *f_y_* is the yield strength for steel (691 MPa), *f_u_* is the ultimate tensile strength for FRP (1100 MPa for GFRP, 2100 MPa for CFRP), and *τ*_max_ is the maximum bond stress from fitted *τ–s* models.

The calculated values (169.5 mm for steel, 2291.7 mm for GFRP, and 8181.8 mm for CFRP) were obtained under the simplifying assumption of constant bond stress equal to *τ_max_* along the embedment length. Therefore, the computed *l*_*b*,*eff*_ values should be interpreted solely as configuration-specific comparative indicators rather than design anchorage lengths. Despite this simplification, the results clearly illustrate the substantial difference in bond capacity between ribbed steel and smooth FRP bars embedded in the same hybrid-fiber HPC matrix. The 200 mm embedment length was sufficient for steel to mobilize its mechanical resistance, whereas FRP bars were unable to develop their tensile capacity under the tested configuration.

These findings provide a practical basis for selecting bond–slip relationships in nonlinear structural analyses and indicate that smooth FRP bars require modified anchorage strategies (e.g., increased development length or surface enhancement) to achieve adequate performance in hybrid-fiber HPC members.

## 5. Limitations

This study focuses on a single hybrid-fiber HPC mixture (steel–basalt–polypropylene ≈ 1 vol.% total fiber content), tested at 28 days under uniform curing, with one embedment length (200 mm) and ϕ12 mm bars. Consequently, matrix composition, bar diameter, surface conditioning (e.g., sand-coating), and embedment-length effects were not investigated parametrically.

The sample size in flexural and pull-out series was limited; although *τ–s* curves and bond energies (*G_b_*) were processed using consistent statistical procedures, a broader quantification of scatter and reliability would require larger experimental cohorts.

Slip was approximated from actuator displacement with system-compliance correction. While this approach ensured internally consistent comparison across steel, GFRP, and CFRP reinforcement, direct slip measurements using LVDTs or digital image correlation (DIC) at the loaded or free end would further reduce uncertainty, particularly in the small-slip regime.

Finally, the constitutive formulations (ramp–softening for steel and ramp–plateau/exponential for FRP) were calibrated exclusively to the present dataset. Their applicability to other hybrid-fiber HPC compositions, fiber dosages, or FRP surface textures should therefore be considered configuration-dependent.

## 6. Conclusions

This study provides a configuration-consistent experimental comparison of ribbed steel and smooth FRP (GFRP, CFRP) bars embedded in the same hybrid steel–basalt–polypropylene fiber high-performance concrete matrix under identical curing and geometric conditions. The adopted unified framework enables direct assessment of interfacial mechanisms without confounding matrix variables.

The hybrid-fiber HPC achieved *f_c_* ≈ 82 MPa with balanced tensile response, and high fracture energy *G_f_* ≈ 3.71 kJ/m^2^, confirming stable post-peak crack bridging associated with multi-scale fiber synergy.

Bond behavior differed fundamentally by reinforcement type. These differences reflect the dominant interfacial mechanisms governing force transfer. In ribbed steel bars, bond is controlled by mechanical interlock and confinement effects, which enable progressive stress redistribution and high energy dissipation. In contrast, smooth FRP bars rely primarily on adhesion–friction mechanisms, which provide limited resistance to slip and result in reduced bond capacity and energy absorption. Ribbed steel developed *τ*_max_ ≈ 13.05 MPa with pronounced interlock-driven response and high bond energy (~146 N/mm), whereas smooth FRP bars exhibited significantly lower *τ_max_* ≈ 1.46 MPa for GFRP and 0.78 MPa for CFRP, and adhesion–friction-controlled *τ–s* behavior with limited energy dissipation (≈3–4 N/mm).

The normalized bond strengths *τ_max_*/√*f_c_* for FRP reinforcement fall within reported ranges for high-performance matrices, while the steel bar significantly exceeds FRP due to mechanical interlock and confinement within the hybrid fiber matrix.

Calibrated bond–slip constitutive descriptions (ramp–softening for steel and ramp–plateau for FRP) accurately reproduced the experimental *τ–s* hierarchy and provide design-oriented parameters for anchorage assessment and nonlinear FE modeling of hybrid-fiber HPC members with mixed reinforcement systems.

Although based on a controlled experimental program, the results establish a mechanistically coherent reference dataset for evaluating bond performance in hybrid-fiber HPC systems and highlight both the advantages and limitations of smooth FRP reinforcement in durability-driven or hybrid structural applications.

From a structural perspective, the results indicate that while ribbed steel reinforcement can effectively utilize the enhanced confinement provided by hybrid fibers, smooth FRP bars require either increased embedment length or surface modification to achieve comparable anchorage performance. This distinction is critical for the design of hybrid or durability-oriented reinforced concrete elements.

## Figures and Tables

**Table 1:** Physical, mechanical, and chemical properties of CEM I 42.5 R cement.

Property	Unit	Value
Setting time		
–Initial	min	190
–Final	min	250
Standard consistency (water demand)	%	28
Specific surface area (Blaine)	cm^2^/g	3985
Mechanical properties		
Compressive strength (2 days)	MPa	30.4
Flexural strength (2 days)	MPa	5.41
Chemical composition		
SiO_2_	%	20.46
Al_2_O_3_	%	4.15
Fe_2_O_3_	%	3.37
CaO	%	65.1
MgO	%	1.21
SO_3_	%	2.58
Na_2_O	%	0.24
K_2_O	%	0.46
Cl^−^	%	0.091
Insoluble residue	%	0.29
Loss on ignition	%	3.44

**Table 2:** Physical and mechanical properties of fibers used in the hybrid-fiber HPC mixture.

Fiber Type	Shape	Length (mm)	Diameter (mm)	Density (g/cm^3^)	Elastic Modulus (GPa)	Tensile Strength (MPa)
Steel (ST)	Hooked-end	50	1	7.8	200	1100
Basalt (BF)	Flat bundle (13 µm filaments)	12	0.013	2.7	70	1700
Polypropylene (PP)	Fibrillated	12	0.03	0.9	3.5	350

**Table 3:** Mechanical properties of reinforcing bars.

Bar Type	Surface	Diameter (mm)	Tensile Strength (MPa)	Elastic Modulus (GPa)	Ultimate Strain (%)	Density (g/cm^3^)
Steel B500SP	Ribbed	12.0	691	203	33	7.85
GFRP	Smooth	12.0	1100	50	2.2	2.10
CFRP	Smooth	12.0	2100	160	1.3	1.60

Note: Values for GFRP and CFRP bars correspond to manufacturer data (nominal range for 12 mm bars), while steel values were obtained experimentally on bars from the same batch.

**Table 4:** Mix proportions of the hybrid fiber-reinforced HPC per 1 m^3^.

Component	Quantity [kg/m^3^]
Cement CEM I 42.5 R	745
Granite aggregate (2–8 mm)	660
Granite aggregate (8–16 mm)	330
Quartz sand (0–2 mm)	500
Water	210
Superplasticizer (BASF Glenium SKY 591)	20 L
Steel fibers (ST)	25.7
Polypropylene fibers (PP)	3.0
Basalt fibers (BF)	8.7

**Table 5:** Summary of mechanical properties (mean, SD, COV, and 95% CI).

Property	Symbol	Unit	Mean	SD	COV [%]	95% CI
Compressive strength	*f_c_*	MPa	81.98	0.20	0.24	[81.73, 82.23]
Splitting tensile strength	*f*_*ct*,*sp*_	MPa	5.00	0.14	2.8	[4.88, 5.12]
Flexural strength (3-pt)	*f*_*ct*,*flex*(3*pt*)_	MPa	6.72	0.08	1.2	[6.65, 6.79]
Flexural strength (4-pt)	*f*_*ct*,*flex*(4*pt*)_	MPa	5.98	0.04	0.7	[5.94, 6.02]

**Table 6:** Fracture parameters obtained from the load–deflection response of the notched hybrid-fiber HPC beam.

Parameter	Symbol	Unit	Value
Maximum load	*P_max_*	kN	2.981
Deflection at *P_max_*	*δ_max_*	mm	0.805
Residual load ratio at *δ* = 0.5 mm	*R*_0.5_	–	0.994
Residual load ratio at *δ* = 1.0 mm	*R*_1.0_	–	0.994
Residual load ratio at *δ* = 1.5 mm	*R*_1.5_	–	0.968
Fracture energy	*G_f_*	J/m^2^	3706.75

**Table 7:** Bond parameters for steel, GFRP, and CFRP bars.

Bar	τ_max_ [MPa]	s(τ_max_) [mm]	k_0_ [MPa/mm]	G_b_ [N/mm]
CFRP	0.78	0.79	11.65	3.04
GFRP	1.46	1.85	1.80	3.78
Steel	13.05	13.10	0.44	145.94

**Table 8:** Model parameters for *τ–s* fits.

Bar Type	Model	τ_max_ [MPa]	k_0_ [MPa/mm]	s_1_ [mm]	k_1_ [MPa/mm]	R^2^	RMSE [MPa]
Steel	Ramp–Softening	12.23	3.18	3.85	0.048	0.988	0.47
GFRP	Ramp–Plateau	1.44	1.49	0.97	–	0.994	0.03
CFRP	Ramp–Plateau	0.77	1.5	0.52	–	0.998	0.006

## Data Availability

The original contributions presented in this study are included in the article. Further inquiries can be directed to the corresponding author.
